# Auditory discrimination in aging bilinguals vs. monolinguals with and without hearing loss

**DOI:** 10.3389/fragi.2023.1302050

**Published:** 2024-01-11

**Authors:** Miwako Hisagi, Beatriz Barragan, Arlene Diaz, Kai White, Margaret Winter

**Affiliations:** ^1^ Department of Communication Disorders, Doctor of Audiology Program, California State University-Los Angeles, Los Angeles, CA, United States; ^2^ Department of Audiology and Speech-Language Pathology, AT Still University, Mesa, AZ, United States

**Keywords:** aging, bilingualism, hearing loss, vowel discrimination, speech perception in noise

## Abstract

Demands for effective assessments of speech perception specific to the aging brain are increasing, as the impacts of hearing loss on an individual’s functional health, socialization, and cognition have become more widely recognized. Understanding the mechanisms behind the optimal function of the aging brain in relation to speech and language is challenging, especially in the bilingual population where the language learning and language interference processes could be mistaken for perceptual difficulty. Age-related presbycusis is unavoidable, and the contributions of this sensorineural hearing loss (SNHL) process on impaired speech recognition are not completely understood. This lack of understanding of the effects of aging and bilingual language competency on speech perception can act as a barrier to successful auditory rehabilitation. The present study investigated the effects of aging on vowel sound discrimination in adult listeners (age 50+) with the following characteristics: American English (AE) monolinguals with normal hearing, simultaneous or early sequential Spanish-English (SE) bilinguals with normal hearing, and AE monolinguals with SNHL (AE-SNHL). The goal was to identify the differences in vowel sound discrimination performance between the monolingual and bilingual aging populations to guide future language assessments and intervention processes. English vowel discrimination was assessed using an AXB discrimination task in quiet and using the Quick Speech in Noise (QuickSIN) test. SE bilinguals were outperformed by AE and AE-SNHL monolinguals, suggesting SE bilinguals primarily use their L1 acoustic properties to discriminate speech segments. No significant difference was found in QuickSIN performance between the bilingual and the monolingual groups, but there was a significant difference between AE and AE-SNHL. In conclusion, vowel discrimination was affected by interference with the native language, while performance in the noise condition was affected by hearing loss. The results of this study contribute to our understanding of the age-related speech processing deficits from three different aging groups regarding the cognitive control system.

## 1 Introduction

Aging can impact an individual’s functional health and ability to comprehend and produce language. Effects include reduced processing speed (Aerts et al., 2013), increased difficulty comprehending complex sentences, reduced capacity of working memory, more difficulty with word recall (Rossi and Diaz, 2016), difficulty in phoneme categorization and discrimination (Mattys and Scharenborg, 2014), more challenges moving articulators into position (i.e., neutralizing vowels), and more trouble processing degraded signals (Van Engen et al., 2020), among others. Age-related decline of speech-responsive brain regions in the temporal lobe appear to result in a reliance on frontal lobe regions associated with cognitive control systems to recognize speech (Eckert et al., 2008); therefore, a preserved cognitive control system would better support speech recognition in older adults.

The cognitive reserve is an individual’s cognitive neural processing ability that is beyond the minimal capacity necessary to function, which allows for coping with changes as a result of the aging brain (Perneczky et al., 2019; Stern 2012). This reserve may not be used under typical conditions, but it is available when demands are high. The cognitive reserve allows individuals with a neurological pathology, or simply those who are experiencing age-related changes in the brain, to maintain cognitive performance, despite degradation within their brain (Abutalebi, et al., 2015). Individuals with more cognitive reserve are able to cope with neurological changes that occur due to aging or pathology; therefore, they are more likely to age without losing as much cognitive performance as those with less reserve. Older individuals who have significant cognitive reserve are more likely to maintain better performance on tasks requiring executive functions. For example, a speech in noise test requires focusing on the speech while ignoring the noise, which demands a large amount of inhibition and working memory especially when the person has a hearing loss, which is common with aging. The cognitive reserve will support performance on this task, allowing for the recruitment of additional brain areas to process the challenging auditory signal (Abutalebi, et al., 2015).

Interestingly, the cognitive reserve may be increased by speaking additional languages (Kroll and Dussias, 2017; Mukadam et al., 2017). It is estimated that half of the global population is capable of speaking and comprehending more than one language (Mathews, 2019). Multiple studies have suggested that bilingual individuals, compared to monolinguals, performed at a higher level on some cognitive tasks such as visual-spatial skills, metalinguistic awareness, selective attention, working memory, critical thinking, and cognitive flexibility (Bialystok, 2010; Bialystok et al., 2012; Costa and Sebastián-Galles, 2014; Bialystok et al., 2016; Kroll and Dussias, 2017; Mukadam et al., 2017). It is hypothesized that this bilingual advantage is a consequence of the need of the bilingual individual to develop skills to reduce language processing conflicts between their two activated languages.

Early stages of bilingual brain development involve the refinement of the language(s)’ phonetic perception, achieved through the formation of prominent phonetic prototypes (e.g., Kuhl et al., 2014). These prototypes enable the brain to interpret and derive meaning from the acoustic information of the language, eventually contributing to the development of the phonological system. Continuous exposure to the phonetic information of a language leads the brain to fine-tune itself to the specific acoustic characteristics of that language’s phonemes, creating cognitive phonetic categories. Phonemes that do not fit within these established categories are assimilated into the closest matching category and are therefore not distinctly discriminated. Peltola et al. (2006) explored phoneme perception of Finnish-native children and found that when presented with vowel stimuli absent in their native language, children assimilated foreign vowels into their existing phonetic prototypes, making foreign sounds discrimination difficult for them. Other studies have aimed to understand the processes underlying the brain’s ability to manipulate and perceive information in different types of bilinguals, such as early bilinguals, late bilinguals, and proficient sequential bilinguals who acquired their second language through schooling (Allik et al., 1998). Biagorri et al. (2019) looked at the ways in which language systems of bilinguals interact behaviorally and physiologically during various tasks and found that early second language (L2) learning enhances the ability to perceive cross-language phonetic differences, despite the continuous influence of the first acquired language (L1) on L2 perception.

The Perceptual Assimilation Model (PAM) is based on second language learners’ ability to perceive speech sounds in their L2 during the acquisition process (Best, 1995; Best and Tyler, 2007; Tyler et al., 2014). The PAM suggests that the brain categorizes speech sounds from L1 and L2 into distinct speech categories. The PAM offers a framework for predicting the ease with which non-native listeners discern the contrast between two phonemes. PAM evaluates the phonetic resemblance of L2 speech sounds to L1 categories, forecasting whether these L2 sounds will be perceived as either good or poor examples within an L1 category. For example, according to this model it is anticipated that English [ɑ], [ʌ], and [æ] will all be incorporated into the Spanish/a/. Additionally, there is some indication that English [ɑ] is a superior exemplar of Spanish/a/compared to [ʌ] or [æ] (Shafer et al., 2022). Moreover, the ability to discriminate may hinge on the duration for which a particular stimulus must be retained in memory, especially the first stimulus in a sequence (Cowan and Morse, 1986). The process of categorizing L1 and L2 sounds involves comparing and discriminating between L1 and L2 speech sounds, as well as discriminating among L2 speech sounds.

According to Fledge and Strange, (1995), the age of L2 acquisition influences the refinement of a bilingual individual’s L2 phonological perception. Hisagi and others (2020), evaluated the perception of English vowels [ɑ] (as in “hot”), [ʌ] (as in “hut”), and [æ] (as in “hat”) from younger adults (19–39 years) of Spanish-English (SE) bilinguals whose L1 is Spanish and Japanese-English bilinguals whose L1 is Japanese. The aim of the study was to evaluate the extent to which bilingual individuals rely on the L1 acoustical characteristics to discriminate L2 vowels. American English (AE) vowels differ in intrinsic vowel duration (Peterson and Lehiste, 1960), and spectrally between “long” vs. “short” adjacent vowels (i:- ɪ, e:- ɛ, æ:- ɛ, ɑ:- ʌ, u:- ʊ). Japanese vowels differ exclusively in duration as in/i, i:, e, e:, a, a:, o, o:, u, u:/., therefore, Japanese listeners rely heavily on durational cues. On the other hand, Spanish does not have a durational cue in the vowel system, therefore, according to the PAM model (Best and Tyler, 2007) English [ɑ], [ʌ], and [æ] are all assimilated into Spanish/a/. Results showed that early bilinguals compared to late bilinguals performed better in an L2 vowels discrimination task.

In contrast to the PAM model, the Automatic Selective Perception (ASP) model describes a more active approach to perceiving L1 and L2 speech information, emphasizing the brain’s active collection of acoustical-phonetic parameters of L1 through habitual exposure, while L2 requires more cognitive effort to differentiate the speech sounds. The ASP model (Strange, 2011), presents a framework for understanding how individuals acquire their L1 and L2. According to the model, individuals engage in selective perceptual learning routines (SPRs) where they focus on and retain relevant acoustical and meaningful linguistic information specific to their L1, establishing expertise in that language. A native language’s SPRs is an extensively practiced sequence formed as individuals acquire their first language (L1) skills (Strange, 2011; Strange, 2011). These ingrained routines assist listeners in capturing vital perceptual-acoustic signals (including tone, duration, or spectral cues) essential for swift comprehension of spoken language. By doing so, this process alleviates the cognitive burden associated with speech perception, freeing up cognitive resources for enhanced language understanding. ASP refers to the consistent exposure to a language, which allows the individual to habituate to the phonetic and phonological properties of that language, including spectral and temporal characteristics (Strange, 2011; Hisagi et al., 2020). During phonological acquisition, children execute and rehearse these SPRs allowing the brain to solidify sensitivity to their language’s phonetic properties, such as spectral characteristics, and promote automaticity. Phonemes not processed through these routines are assimilated and therefore not perceived distinctly. More attention and focus are required for acquiring L2, as individuals must extract information to differentiate phonetic contrasts that do not exist in their L1. In theory, L1 SPRs solidify during early development. In contrast, L2 learners must allocate attentional resources to effectively discern the pertinent L2 perceptual-acoustic cues, requiring them to override their established L1 SPRs. Consequently, speech perception in the L2 demands heightened cognitive exertion, reflected in prolonged reaction times and diminished accuracy on speech perception tasks. Individuals who become bilingual early in life may cultivate SPRs for both languages that mirror those observed in a single L1. An inquiry at hand explores whether early bilinguals exhibit speech perception comparable to monolingual listeners, particularly in terms of discrimination accuracy.

Behavioral tasks have been used to measure L2 language processing and automaticity through discrimination tasks. Literature suggests that non-native vowel contrasts with temporal cues may be easier to discriminate than those with spectral cues (Strange, 2011). Hisagi and Strange (2011) investigated the discrimination performance of English monolinguals regarding Japanese vowel, consonant, and syllable contrasts. The study assessed how well AE monolinguals could discriminate Japanese vowels, which differ phonologically in temporal characteristics, when presented in connected speech material. The results indicated that AE monolinguals were able to discriminate Japanese vowel contrasts above chance levels.

The speech discrimination abilities of SE bilinguals in noisy environments have also been evaluated. Mendel and Widner (2015) examined the speech perception abilities of SE bilinguals compared to monolingual English speakers. The study found that bilingual individuals without hearing loss exhibited a mild signal-to-noise-ratio (SNR) loss similar to that of monolinguals with hearing loss. The authors suggested that the bilinguals’ poor performance may be attributed to decreased automaticity in their L2. For example, the words beat-bit (/i/-/ɪ/) sound the same to Spanish listeners with reduced exposure to English as a second language, therefore, more cognitive effort is required to differentiate these speech sounds in L2.

Sensorineural hearing loss (SNHL) is a common condition associated with aging. The manifestations of age-related SNHL, presbycusis, in older adults can be subtle; nonetheless, the broader magnitude of presbycusis may have a direct bearing on the global health of the elderly (Helzner et al., 2005). Age-related SNHL is associated with poorer cognitive functioning and dementia. Individuals with mild, moderate, and severe hearing loss have a 2-, 3-, and 5-fold increased risk, respectively, of developing dementia as compared to individuals with normal hearing (Lin et al., 2011). SNHL affects speech perception in a complex, nonlinear manner due to four characteristics: reduced sensitivity, abnormal growth of loudness (recruitment), reduced frequency selectivity, and reduced temporal resolution. The effects of age-related SNHL processes on impaired speech recognition are not completely understood, and their impact on the bilingual population and their interaction with the cognitive reserve have been minimally studied. This lack of understanding of the effects of aging on speech perception can act as a barrier to successful auditory rehabilitation in bilinguals.

There has been an increase in the number of bilingual aging 60+ respondents to the U.S. Census from 12.6% to 15.7% between the years 2000 and 2019, of whom 5.9 million (50% of the population 60+ who speak a language other than English at home) are Spanish-English speakers (Dietrich and Hernandez, 2022). A percentage of this population is expected to have language difficulties, as aging is an important risk factor for language comprehension decline (Peelle, 2019), as is hearing loss (Peelle and Wingfield, 2016). Understanding the factors influencing language processing in this population is essential towards developing strategies to prevent, assess, and intervene regarding language comprehension decline. Improving such assessment and intervention would improve functional health.

The present study investigated the effects of aging on vowel discrimination in monolingual older adults with and without hearing loss, in comparison to bilingual older adults with normal hearing. It is relevant to identify the differences in linguistic discrimination performance between the monolingual and bilingual aging populations to guide the development of language assessments and intervention processes. Specifically, this study explored in older populations, i) the vowel discrimination differences between monolingual and bilinguals, ii) the effect of background noise in vowel discrimination in monolinguals and bilinguals, and iii) the effect of SNHL on vowel discrimination in monolinguals.

## 2 Methods

### 2.1 Participants

The present study recruited adult (50+ years) American English (AE) monolinguals and Spanish-English (SE) bilinguals. The sample was divided into the following groups: Group 1—AE monolinguals with normal hearing (*n* = 18); Group 2—SE bilinguals with normal hearing who acquired Spanish as their native language and English later during childhood (*n* = 18); and Group 3—AE monolinguals with SNHL (*n* = 10). Monolingual participants had minimal experience with any foreign language and spoke only English in their daily activities. Participants were recruited on a volunteer basis via a self-selection sampling from the local community and were gathered from the Southern California region via California State University Los Angeles’ social media outlets, flyers, and word of mouth. Participants were screened for negative histories of neurological pathologies and otorhinolaryngological surgeries, as well as normal otoscopic examination findings. Participants with any history of neurological disease or psychiatric syndrome were excluded from this study. Table 1 describes the participant’s information for each group.

**TABLE 1:** Participants’ Demographic and Language Background Information. *Note*. Descriptive statistics showing group averages, including mean age with range, gender distribution (F = female; M = male), mean self-rated English proficiency across all four language domains, percent of time English were used at home before age 5, and mean age of self-reported English acquisition with range.

	AE	SE	AE-SNHL
	American English monolinguals	Spanish-English bilinguals	American English monolinguals with sensorineural hearing loss
Age	58 yr (SD = 8.22)	61.3 yr (SD = 8.87)	65 yr (SD = 11.30)
	Range (50–78)	Range (50–82)	Range (53–85)
Gender	11F 7M	14F 4M	4F 6M
Mean (four linguistic domains)	9.76	7.26	9.77
% Of English used at home before age 5	95%	18%	95%
Age of English Acquisition	6 months (0∼3 years)	6.5 yr (1–13 years)	6 months (0∼4 years)

Participants self-reported information about their language background and language proficiency level through an online Qualtrics generated survey, similar to the one previously used in Hisagi et al. (2020). The survey consisted of several questions including ratings for the perceived level of English language expertise using a Likert Scale across four language domains: reading, writing, speaking, and speech understanding. A rating of 0 represented no proficiency whereas a rating of 10 indicated ‘native-like’ proficiency. Overall, SE bilinguals rated their English proficiency lower than AE monolinguals.

Most participants in Group 3 had symmetrical hearing loss between ears. One participant had mild sloping to moderately-severe SNHL in the right ear and mild sloping to severe SNHL in the left ear. Two participants had mild sloping to moderate SNHL in the right ear and mild sloping to severe SNHL in the left ear. Table 2 describes the type and degree of hearing loss in Group 3 participants.

**TABLE 2:** Group 3 Hearing Loss Characteristics. *Note*. Degree and shape of hearing loss by number of ears, left (L) and right (R), for Group 3 participants.

Number of ears (Right/Left)	Degree of hearing loss
6 (2R 4L)	Flat Mild
8 (5R 3L)	Mild sloping to moderate
1 (1R)	Mild sloping to moderately-severe
1 (1L)	Mild sloping to severe
4 (2R 2L)	Mild sloping to profound

### 2.2 Stimuli

The AXB discrimination task used in this study was identical to Hisagi et al. (2020). The English vowels [ɑ] (as in “hot”), [ʌ] (as in “hut”), and [æ] (as in “hat”) were used. The pre-recorded stimuli were produced by a young male native speaker of a New York dialect of AE with a fundamental frequency of 150 Hz (SD of 5 Hz), recorded in a sound booth at a sampling rate of 22,050 Hz. The vowels [æ ɑ ʌ] were produced in citation form in the nonsense syllable form [Vpə], where “V” is the target vowel. There were three tokens for each vowel. The mean word form duration was 427 ms ([æpə]), 392 ms ([ɑpə]), and 375 ms ([ʌpə]). The mean vowel duration was 187 ms ([æ]), 184 ms ([ɑ]), and 134 ms ([ʌ]), with a long-to-short vowel ratio of 1.4. The range for the fundamental frequency is 126–137 Hz for [ɑ], 126–131 Hz for [æ], and 130–136 Hz for [ʌ] with a mean of 132 Hz. The stimulus level was normalized using root mean square. To allow the participants to select a comfortable presentation level, they were presented with sample stimuli matching the duration and intensity of the experimental stimuli before beginning the experiment and were asked to adjust the sound intensity to a comfortable level. Therefore, the intensity of delivery varied across participants.

### 2.3 Instruments and software

A Grason-Stadler GSI Audiostar Pro audiometer calibrated according to ANSI (S3.6-1969) specifications was used for all audiologic evaluations. Grason-Stadler TympStar immittance equipment was used to obtain tympanograms and ipsilateral acoustic-reflex thresholds. Participants completed the experimental AXB listening task using E-Prime software on a Dell laptop running on Microsoft Windows operating system.

The Quick Speech in Noise (QuickSIN) test estimates signal-to-noise ratio (SNR) loss. The test uses a list of six sentences with five key words per sentence, presented embedded in four-talker babble noise. The first sentence starts with 25 SNR (considered very easy) and the last sentence presented with 0 SNR (considered extremely difficult), decreasing in 5-dB steps (i.e., 25, 20, 15, 10, 5 and 0). Table 3 describes the norm for this test.

**TABLE 3:** QuickSIN SNR Loss Norms. *Note*. QuickSIN signal to noise ratio (SNR) loss norms (Etymotic Research Inc., 2006).

0–3 (dB)	No SNR loss
3–7	Mild SNR Loss
7–15	Moderate SNR Loss
>15	Maximum SNR Loss

### 2.4 Procedures

After reading and signing the informed consent, prepared in accordance with the Declaration of Helsinki and approved by the California State University Los Angeles Institutional Review Board (IRB), participants completed a background questionnaire via the Qualtrics online platform. All procedures took place in a sound treated booth. Participants completed a full audiological evaluation including otoscopy, immittance, air conduction, bone conduction, speech recognition testing using spondees, word recognition testing using recorded Northwestern University Auditory Test No.6 (NU-6) word list (Auditec, Inc.), and QuickSIN (Etymotic Research Inc, 2006) testing which uses low-context sentences recorded in four-talker babble to estimate SNR loss. Participants received a pure tone threshold evaluation in each ear at 250, 500, 1,000, 2000, 3,000, 4,000, 6,000, and 8,000 Hz utilizing a modified version of the Hughson-Westlake method.

The AXB discrimination task was conducted last. No stimuli were permitted to reach sound levels that are potentially hazardous to hearing sensitivity. Participants heard a sequence of three/Vpə/nonsense syllable stimuli and were asked to categorize whether the second, target stimulus (X) was more similar to the first stimulus (A) or the third stimulus (B). The middle stimulus (X) matched either the A or B stimulus in terms of target vowel. They were instructed to press “d” on their keyboard if the middle stimulus (X) was more similar to the first stimulus (A) or “k” if the middle stimulus was more similar to the final stimulus (B). Participants were given two practice blocks to familiarize them with the task and allow them to adjust the volume of the stimulus. The first practice block included 6 trials that were normalized to have an identical root-mean-square value as the experimental stimuli and provided information about the accuracy of participant responses. The second practice block included 10 trials without feedback about response accuracy. The stimuli for the practice trials followed the same characteristics as the experimental stimuli. After the practice blocks, participants listened to three experimental blocks and were encouraged to take a short (2–5 min) break between block presentations.

Each of the three blocks contained stimuli with one target vowel, with the presentation order of the stimuli within each block in addition to the presentation order of the blocks being randomized. Each block contained four different stimulus combinations based on the non-target stimulus vowel and the presentation order, e.g., **[a] [a]** [ʌ], **[a] [a]** [æ], [ʌ] [**a**] [**a**], [æ] **[a] [a]**. Each of the three experimental blocks contained 72 trials (3 different tokens per vowel x 4 combinations x 6 repetitions = 72 trials), for a total of 216 experimental trials per participant. The full list of stimulus combination types are represented in Table 4. The entire experimental session lasted approximately 1.5 h.

**TABLE 4:** Twelve AXB Discrimination Categories. *Note.* This table illustrates all possible combination types in the experimental blocks. **A** represents the first vowel presented, **X** represents the middle or target vowel, and **B** represents the last vowel presented in the sequence.

	Combination types		A	X	B
Block A	1	LL-S	[ɑ:]	[ɑ:]	[ʌ]
	2	LL-L	[ɑ:]	[ɑ:]	[æ:]
	3	S-LL	[ʌ]	[ɑ:]	[ɑ:]
	4	L-LL	[æ:]	[ɑ:]	[ɑ:]
Block B	5	SS-L	[ʌ]	**[ʌ]**	[ɑ:]
	6	SS-L	[ʌ]	**[ʌ]**	[æ:]
	7	L-SS	[ɑ:]	**[ʌ]**	**[ʌ]**
	8	L-SS	[æ:]	**[ʌ]**	**[ʌ]**
Block C	9	LL-S	[æ:]	[æ:]	[ʌ]
	10	LL-L	[æ:]	[æ:]	[ɑ:]
	11	S-LL	[ʌ]	[æ:]	[æ:]
	12	L-LL	[ɑ:]	[æ:]	[æ:]

## 3 Results

### 3.1 Overall group comparison

**FIGURE 1:**
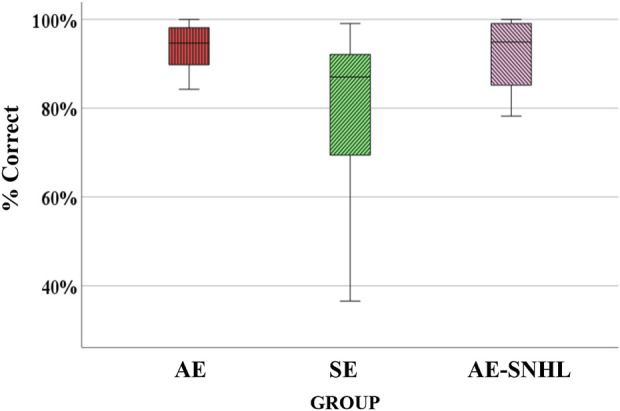
Group comparison for overall accuracy. American English monolinguals (AE); Spanish-English bilinguals (SE); American English monolinguals with sensorineural hearing loss (AE-SNHL). The box-and-whiskers bars represent median (horizontal line in the box) with interquartile range (upper and lower quartiles). Whiskers represent upper and lower extremes.

Overall discrimination accuracy across the three groups was assessed. Because the variances were not homogeneous, Welch’s F test was used for group comparisons. Results showed significant differences among groups [F_welch_ (2, 20.762) = 4.575, *p =* .023]. Post-hoc Tukey (Kramer’s) HSD showed significant differences between the AE group and SE group (*p* = .005), where AE participants performance was better than those in the SE group, and between SE and AE-SNHL groups (*p* = .037), where AE-SNHL participants performance was better than those in the SE group. A boxplot of the overall accuracy per group is shown in Figure 1. Observed power (*post hoc* power) analysis revealed a power exceeding 0.80 [F (2, 44) = 6.279, *p* = 0.04, observed power = 0.875].

### 3.2 Group comparison by block

**FIGURE 2:**
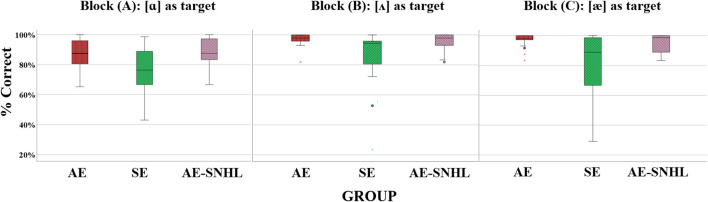
Group comparison for accuracy by block. American English monolinguals (AE); Spanish-English bilinguals (SE); American English monolinguals with sensorineural hearing loss (AE-SNHL). The box-and-whiskers bars represent median (horizontal line in the box) with interquartile range (upper and lower quartiles). Whiskers represent upper and lower extremes. Block B has short vowel in the middle and long vowel on the sides. Block A and C middle is long vowel so it is compared with other long vowels and short vowels but at least two long vowels.

Welch F test (equal variances not assumed) was used to determine the differences among the three stimulus blocks. Block A presented [ɑ] as the target, block B presented [ʌ] as the target, and block C presented [æ] as the target. Discrimination accuracy by block was evaluated and the following results were found: Block A and C were significantly different among groups [Block A: F_welch_ (2, 23.568) = 4.449, *p* = 0.023 and Block C: F_welch_ (2, 21.049) = 3.740, *p* = 0.041], while Block B showed no significant difference between groups [F_welch_ (2, 20.202) = 2.663, *p* = 0.094]. Post-hoc Tukey (Kramer’s) HSD showed that in block A the AE group performed significantly better than the SE group (*p* = 0.012), the same as the AE-SNHL performance over the SE group (*p* = 0.04). In block C, only the AE group performed significantly better than the SE group (*p* = 0.011). A boxplot of group accuracy by block is shown in Figure 2.

### 3.3 Combination descriptive analysis

Based on previous research (Best and Tyler, 2007; Hisagi et al., 2020) it was predicted that contrasts with spectral differences like [ʌ] vs. [æ] (combination types 6, 8, 9, 11) would be easier than contrasts with duration differences only like [ʌ] vs. [ɑ] (combination types 1, 3, 5, 7) and [ɑ] vs. [æ] (combination types 2, 4, 10, 12). Table 5 shows descriptive statistics for each combination based on the mean number of errors, standard deviation, and median. Each participant performed in 18 trials per combination in each block.

**TABLE 5:** Descriptive analysis of errors. *Note.* Mean number of errors, standard deviation (SD), and median. AE = American English monolingual group; SE = Spanish/English bilingual group; AE-SNHL = American English monolingual with sensorineural hearing loss group. Bolded (yellow): mean error rate >3.0.

	Block	A				B				C			
		1	2	3	4	5	6	7	8	9	10	11	12
		LL-S	LL-L	S-LL	L-LL	SS-L	SS-L	L-SS	L-SS	LL-S	LL-L	S-LL	L-LL
**AE**	**mean**	0.56	1.39	**3.44**	**3.83**	0.67	0.22	1.11	0.50	0.11	0.11	0.72	1.94
	**SD**	0.984	1.243	3.034	3.167	1.138	0.428	1.491	1.043	0.323	0.323	1.274	2.532
	**median**	0.00	1.00	2.50	4.00	0.00	0.00	0.50	0.00	0.00	0.00	0.00	1.00
**SE**	**mean**	**3.22**	2.94	**4.11**	**6.22**	2.56	2.28	2.83	2.33	2.50	2.50	2.56	**6.17**
	**SD**	4.319	3.351	3.376	5.429	3.989	4.763	3.204	3.290	4.805	3.974	4.232	5.993
	**median**	1.50	1.50	3.50	5.00	1.00	0.00	2.00	1.00	0.00	0.50	1.00	5.50
**AE-SNHL**	**mean**	**3.10**	1.60	2.00	2.00	1.50	0.90	0.50	0.60	0.80	0.00	0.60	2.00
	**SD**	4.630	3.204	2.494	2.055	2.799	1.912	0.972	1.265	1.619	0.000	1.075	3.528
	**median**	1.00	0.00	1.00	1.00	0.50	0.00	0.00	0.00	0.00	0.00	0.00	0.50

We mentioned as “Bolded (yellow): mean error rate > 3.0.” We emphasized the values with bold and yellow highlight.

The SE group produced more errors in Block A (combination types 1, 3, and 4) and Block C (combination type 12) compared to the other combination types. Combination types 1, 3, and 4 also lead to more errors for other groups, although in general they performed better than the SE group. Combination type 4 was the most challenging for AE and SE groups. AE-SNHL performance was in general slightly below the AE group, but better than the SE group. Combination type 1 was the most challenging combination for the AE-SNHL group.

### 3.4 Word recognition NU-6 analysis

**FIGURE 3:**
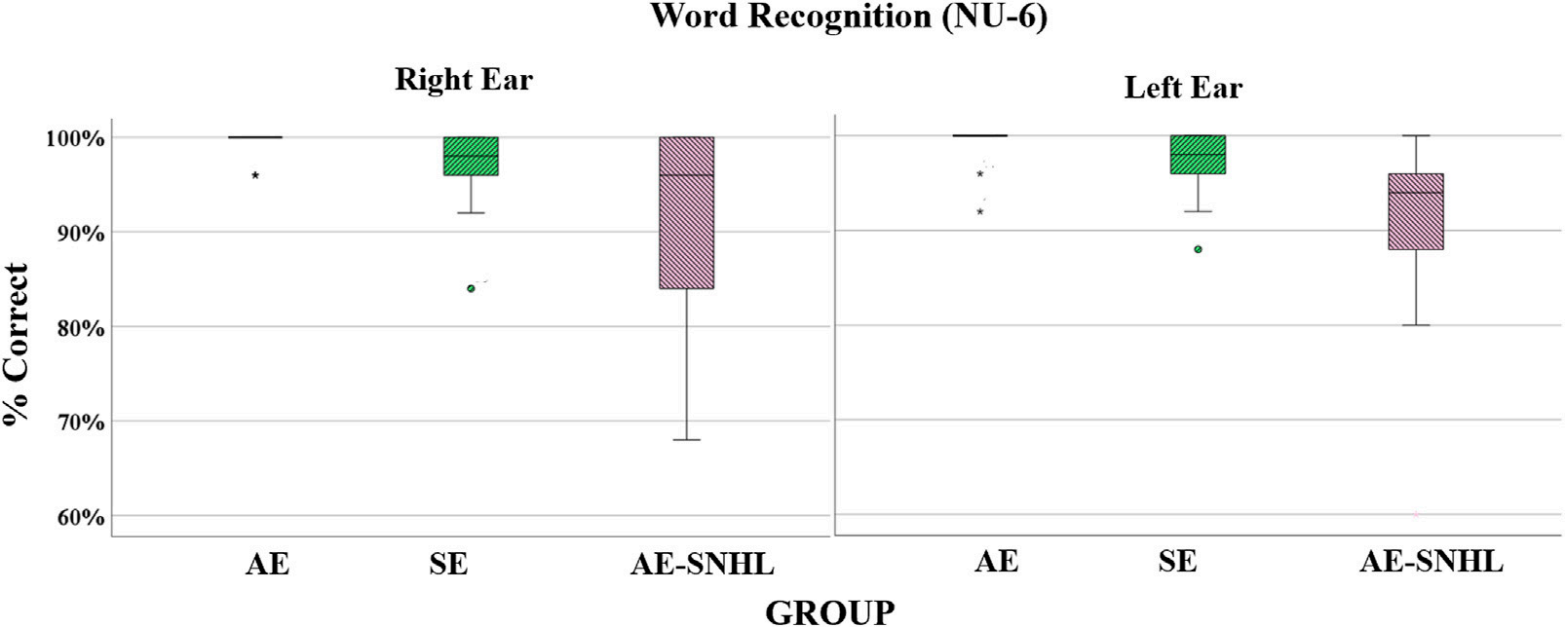
Word recognition accuracy in group comparisons by each ear. American English monolinguals (AE); Spanish-English bilinguals (SE); American English monolinguals with sensorineural hearing loss (AE-SNHL). The box-and-whiskers bars represent median (horizontal line in the box) with interquartile range (upper and lower quartiles). Whiskers represent upper and lower extremes. 25 words x 4 points = 100%.

Welch’s F were used for group comparisons. The results showed significant differences between groups by ear [Right Ear: F_welch_ (2, 17.344) = 4.361, *p =* .029) and (Left Ear: F_welch_ (2, 18.147) = 5.310, *p =* .015]. This result is explained by a significant difference from *post hoc* Tukey (Kramer’s) HSD in word recognition between the AE and AE-SNHL groups (Right *p* = .003 & Left *p* = .001) and between SE and AE-SNHL groups (Right *p* = .035 and Left *p* = .019). A boxplot of the overall accuracy per group is shown in Figure 3.

### 3.5 QuickSIN analysis

**FIGURE 4:**
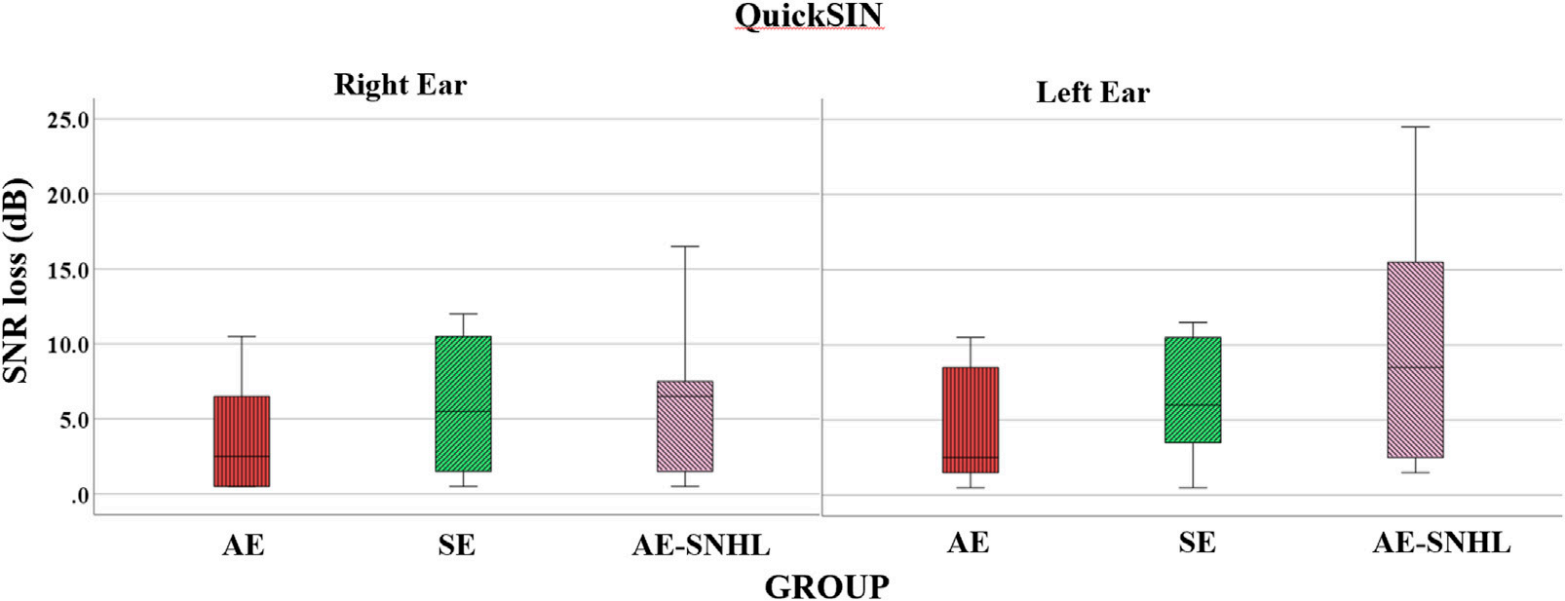
QuickSIN average dB signal to noise ration loss (SNR loss) in group comparisons by ear. Note. American English monolinguals (AE); Spanish-English bilinguals (SE); American English monolinguals with sensorineural hearing loss (AE-SNHL). The box-and-whiskers bars represent median (horizontal line in the box) with interquartile range (upper and lower quartiles). Whiskers represent upper and lower extremes. Lower value = lower noise effect (i.e., better performance).

The average dB SNR loss as calculated by the QuickSIN (see Table 3 above for the norm) for AE group was 3.78 for the right ear and 3.94 for the left ear, indicating no SNR loss. The average for SE group was 5.97 or mild for the right ear and 6.33 or mild for the left ear. The average for AE-SNHL was 6.4 or mild for the right ear and 9.8 or moderate for the left ear. This difference in performance between ears in AE-SNHL may be attributed to the fact that three of the ten participants had asymmetrical hearing loss such that the left ear had a greater degree of hearing loss (see Table 2 above). Welch’s F and t-tests were used for group comparisons. The results showed significant differences among groups only for the left ear [F_welch_ (2, 20.298) = 3.550, *p =* .048], but not right ear [F_welch_ (2, 21.195) = 1.885, *p =* .176]. Post-hoc Tukey (Kramer’s) HSD showed significant differences between the AE group and AE-SNHL groups in the left ear (*p* = .011). A boxplot of the overall accuracy per group is shown in Figure 4.

## 4 Discussion

This study evaluated the differences in linguistic sounds discrimination performance between a AE monolingual aging population with and without SNHL and an SE bilingual aging population. Overall, the results showed that AE monolinguals with and without SNHL performed significantly better than SE bilinguals in the vowel discrimination task. These results suggest that SE bilinguals primarily use their L1 acoustic properties to discriminate speech segments. Similar to Hisagi and others’ (2020) findings, SE bilinguals struggled to discriminate when [a] and [æ] served as target stimulus, suggesting difficulties in using spectral vowel sound differences to help them in the discrimination task. The Spanish/a/is more similar to English [ɑ] than [ʌ] or [æ] (Shafer et al., 2021), which may contribute to the difficulty differentiating the sounds demonstrated by SE bilinguals. According to the PAM (Best and Tyler, 2007), the differences between English and Spanish vowel sounds will result in the assimilation of English [ɑ], [ʌ], and [æ] into the Spanish/a/. Strange (2011) and Fledge and Fledge, (1995) described how L2 selective perception routines require higher attention and cognitive demand to extract L2 acoustic properties, which would interfere with the performance of bilinguals compared with monolingual with or without SNHL.

SE bilinguals’ performance presented more errors in Block A (combination types 1, 3, 4) and Block C (combination type 12), suggesting that bilinguals in this study used more temporal than spectral cues to differentiate the vowel sounds. Discrimination of [ʌ] vs. [æ] (combination types 6, 8, 9, 11) was expected to be easier because of the duration difference, compared to other contrasts like [ʌ] vs. [ɑ] (combination types 1, 3, 5, 7) and [ɑ] vs. [æ] (combination types 2, 4, 10, 12) which is congruent with the performance of all groups, including the bilinguals. One of the highest missed combinations for SE bilinguals was combination type 12, which contained all long vowels requiring spectral knowledge for successfully differentiating them. In the [a]-[æ] contrast as in combination types 1, 3, 9, and 12, there is no durational cue so it is necessary to recognize the spectral cue difference. [æ] is spectrally more distinct than [a], therefore, [æ] is perceptually easier to differentiate than [a]. Thus, combination type 1 and 3 have hard pairs while combination type 9 and 12 have easy pairs. SE bilinguals showed a high number of errors in combination types 1, 3 and 12 suggesting a difficulty in identifying these spectral cues. According to the ASP model, late bilinguals struggle with L2 phoneme perception because they rely more on their L1 SPRs than their L2 SPRs. Most of SE bilinguals in our study fall into the category of late bilinguals since they learned English after the age of 6. Thus, the current findings support this prediction and suggest that the L1 SPRs of AE-monolingual speakers benefit their ability to hear the distinction between these American-English vowels, even after exhibiting symptoms of SNHL, whereas the L1 SPRs of SE-bilingual speakers are not as helpful for distinguishing these vowel pairs, leading to difficulties with both challenging and straightforward pairs.

Memory load may play a role in vowel discriminability, that is, which stimulus needs to be held in memory for a longer period of time (Cowan and Morse, 1986). Based on the previous study by Hisagi et al. (2020), it was expected that the sequence of the AXB presentation could influence the performance. Matching stimulus X with stimulus “B” (e.g., the [ɑ]-**[ʌ]-[ʌ]** order) could produce better scores because they require a shorter time in memory than “A” matching X (e.g., the **[ʌ]-[ʌ]**-[ɑ] order) where longer time is required (Shafer, et al., 2021). However, this memory effect was not found in this study, so memory load was discarded as a potential factor influencing the results.

Previous neurophysiological measures found duration asymmetry, suggesting that duration decrements (short stimuli) are more difficult to process than duration increments (long stimuli) (Shafer et al., 2004; Friedrich et al., 2004; Kirmse et al., 2008). The neurophysiological study by Hisagi et al. (2010) showed that duration increments as frequently repeated sounds were easier to discriminate than duration decrements as frequently repeated sounds (i.e., to discriminate sounds between/tado/vs./taaado/in their study, the train “taado-taado-taado-tado” was easier than “tado-tado-tado-taado” train). That is*, long-long-short* discriminability (i.e., combination types 1 and 9) was easier than *short-short-long* discriminability (i.e., combination types 5 and 6). The likelihood, therefore, is that if the short vowel come first in a string it will be more difficult than if the long vowel comes first. Thus, the sequence of S-L-L requires more effort and more memory than the reverse order (L-L-S). S-L-L (shorter stimuli before longer stimuli) means *hard-easy-easy* processing which requires more processing time at the onset of the presentation while L-L-S means *easy-easy-hard* (longer stimuli before shorter stimuli) and it requires less effort in processing. If the individual has to process the harder stimuli first, uncertainty might draw attention and effort away from the target stimulus, while if they process the easier stimuli first, the certainty level might increase.

Cognitive decline and L2 saliency may have also played a role in discrimination accuracy (Yu et al., 2017). Older populations may experience greater difficulty discriminating vowels due to slower cognitive processes as a consequence of the aging brain. Cortical recruitment and executive function skills play a major role regardless of age. The results from Davies-Venn and Souza’s (2014) study suggest that when audibility is adequately controlled, measuring spectral resolution may identify the listeners who are most susceptible to compression-induced distortions. Cognitive skills such as working memory appear to modulate the negative effect of these distortions for listeners with moderate to severe hearing loss. Although studies such as Mukadam et al. (2017) propose that bilinguals have a cognitive reserve that could be protective against cognitive decline due to aging, other studies show that the bilingual advantage does not translate to better performance in all cognitive tasks. Sorman et al. (2017) found that monolinguals had higher performance on free-recall and retrieving tasks than bilinguals. Saliency could have also affected SE bilinguals’ performance. SE bilinguals reported on average having used English at home only 18% of the time before the age of 5 and the average age of reported English acquisition was 6.5 years. This population may not have had enough English exposure and undergone sufficient selective perception routines to solidify L2 phonetic and phonological characteristics during the optimal time period of language acquisition.

The overall better performance of AE-SNHL over SE bilinguals suggest that vowel discriminability was more affected by the sound knowledge and lack of selective perception routines than by the hearing loss. Participants in the AE-SNHL group were allowed to adjust the task volume to a level that supported their performance, but participants in the SE bilingual group did not have much early exposure to English as their L2 and this condition might impact their performance in non-native language sounds discriminability. Our sample reported a limited use of English at home (18%) before the age 5, and the average age of L2 acquisition was 6.5 years-old, suggesting they learn English at school. Previous research has shown the difficulties in perceiving cross-language phonetic differences and vowel discrimination in late sequential bilinguals (Biagorri et al., 2019; Hisagi et al., 2020). More research in this area is necessary, considering L2 proficiency levels, years of experience with the L2, and percentage of current use and exposure to L2.

QuickSIN results revealed a significant difference in performance between AE and AE-SNHL as expected. But no significant difference in performance between SE and both AE groups was found. It was assumed that monolinguals’ English saliency would give them an advantage in QuickSIN performance, but the SE group performance was not significantly lower than the monolingual groups. This could be an indicator of preserved cognitive ability in the bilingual older group over the monolingual groups, who had the advantage of English language saliency, but it was not enough to perform significantly better than the bilinguals. However, the speech perception ability in noise--as indicated by the QuickSIN performance--affected the vowel discrimination given that performance is governed by many different factors including phonological perception, speech modulation processing, and brainstem processing.

In conclusion, SE bilinguals were outperformed by AE and AE-SNHL monolinguals, especially when long vowels/a/and/æ/were the target stimuli. Their speech perception discriminability was affected by L2 spectral sound vowel differences suggesting SE bilinguals primarily use their L1 acoustic properties to discriminate speech segments, as predicted by the ASP model. On the other hand, there was no significant difference in QuickSIN performance between the bilingual and the monolingual groups, but the significant difference was found between AE and AE-SNHL. Vowel perception was affected by their native language while performance with noise was affected by hearing loss. These results suggest that some discrimination and speech perception errors made by bilingual patients tested via commonly used English word and sentence lists may not reflect their auditory capacity in the usual sense, but rather their ability to discriminate phonemes that are less familiar in their primary language. Therefore, the assessment of word and sentence recognition skills should be tested using the patient’s L1, even when their communication skills in English as their second language, are self-reported as strong or highly proficient.

More data on how cognitive processes are shaped in older bilingual adults could inform the current literature on language processing in non-pathological aging bilingual populations. Examining changes in L2 speech perception gives evidence about auditory neural mechanisms that will help construct more realistic models of L2/bilingual speech perception in terms of brain mechanisms. The contributions of SNHL processes on impaired speech recognition are still not completely understood, but this knowledge is still worth pursuing in the interest of removing barriers to successful auditory rehabilitation. These results should be considered when counseling patients on realistic expectations about amplification.

## Data Availability

The raw data supporting the conclusion of this article will be made available by the authors, without undue reservation.

## Abbreviations

AE, American English; SE, Spanish-English; QuickSIN , Quick Speech in Noise; NU-6, Northwestern University Auditory Test No.6; ASP, Automatic Selective Perception; PAM = =Perceptual Assimilation Model.
